# Editorial Brevities

**Published:** 1887-04

**Authors:** 


### EDITORIALS AND MISCELLANEOUS.

### EDITORIAL BREVITIES.

MACON MEDICAL SOCIETY.—At the annual election of this Society, Dr. E. G. Furguson was chosen President; Dr. H. M. Hatton, Vice-President; Dr. J. Edward Green, Secretary and Treasurer; Dr. R. O. Cotter, Corresponding Secretary; Dr. W. C. Gibson, Reporter; Dr. C. H. Hall, Librarian.

ANNISTON MEDICAL SOCIETY.—From the Alabama Medical and Surgical Journal we learn that the physicians of that thriving town have organized a medical society as above named. At the last meeting, Dr. J. C. LeGrand was elected President, and Dr. T. W. Ayers, Secretary.

ATLANTA MEDICAL COLLEGE.—The graduating exercises of the Atlanta Medical College took place on the night of the 5th ultimo. We have not space for a full account. Rev. Henry Clay Morrison, the well-known and eloquent divine of this city, was the orator of the occasion; Mr. Hooper Alexander, a young and talented lawyer, also of this city, presented the prizes. The valedictory oration was delivered by Dr. J. C. Johnson, of the graduating class. It was a good address. The degree was conferred by the Rev. Dr. Tucker—"*Dominus vobiscum*"!

A DEATH CERTIFICATE.—All applications for free burial in this city must be accompanied by a death certificate, written and signed by the attending physician and indorsed by the City Physician having charge of the Ward in which the deceased lived. The following is a copy of a certificate which was presented the other day to the physician of the Second Ward for his indorsement:

"I sertify that Alice Pain's two twins is ded they was stillbornd."

The indorsement reads as follows: "Correct, except orthography and grammar.

E. v. G., Phys. 2nd Ward."

DEATH OF REV. D. P. SINGLETON.—Mr. Singleton, who had commenced a course of study in the Southern Medical College, visited his home at Norcross, Ga., during the Christmas holidays, where he was taken suddenly ill, and died on the 29th of December, 1886. Mr. Singleton was a young man of good moral character and possessed of many excellent qualities. He connected himself with the Methodist Episcopal Church in 1884, and soon after was licensed to preach the Gospel. As a medical student he was studious and attentive to his duties, and secured the friendship of his associates and the good opinion of the Faculty, who greatly regret his death and warmly sympathize with his relatives in their sad bereavement.

PHYSICIANS ARRESTED.—Two Eclectic Physicians of this city, J. A. Cofer and R. M. Auten, were arraigned before a Justice's Court on the 28th last February for attempting to produce an abortion on a young woman from an adjoining county. They were released on a bond of $500 for their appearance before a higher court. It has not thus far been shown that abortion was produced, and as the girl has since married her seducer it is not likely that this matter will undergo further legal investigation.

THE CASE AGAINST DR. WILE.—On the 30th of last August Dr. Henry Wile, of this city, excised from the arm of a thirteen-year old boy (with the full consent of the latter) some minute grafts to be inserted in an extensive ulcer covering the scalp of a little girl. This exasperated the father of the boy so much that he swore out a warrant against the doctor for assault and battery. The trial, held before Judge Van Epps without a jury, terminated in the complete justification of Dr. Wile, and consequent dismissal of the case against him. We congratulate Dr. Wile upon this happy termination.

COCAINE POISONING.—It would appear, from a paper read by Dr. J. B. Mattison before Kings County Medical Socioty, Brooklyn, N. Y., that cocaine is a remedy that should be used with extreme caution. He reports a number of cases in which serious toxic effects resulted from ordinary doses of the drug, and in one or two instances even death resulted from its use. The toxic symptoms were usually manifested in difficult and oppressed respiration, weak pulse, lips livid, skin pale, and cold, comatose tendency, etc The doctor concludes his interesting paper with the following summary: "Cocaine may be toxic, sometimes deadly in large doses. It may give rise to dangerous or even fatal symptoms in doses usually deemed safe. The danger, near and remote, is greatest when given under the skin. It may produce a diseased condition, in which the will is rendered prostrate and the patient powerless—a true toxic neurosis, more marked and less hopeful than that from alcohol or opium."

